# Impacts of the COVID-19 pandemic on treatment-seeking college students

**DOI:** 10.1080/28324765.2023.2211633

**Published:** 2023-05-21

**Authors:** Natalie R. Pottschmidt, Rebecca A. Janis, Brett E. Scofield, Alaina L. Cummins, Dever M. Carney, Katherine A. Davis, J. Ryan Kilcullen, Hongjun (Michael) Tan, Louis G. Castonguay, Benjamin D. Locke

**Affiliations:** a Department of Psychology, Pennsylvania State University, University Park, PA, USA; b Center for Counseling and Psychological Services, Pennsylvania State University, University Park, PA, USA

**Keywords:** college counseling, mental health, pandemic impact, cohort study

## Abstract

While the impact of the coronavirus disease (COVID-19) pandemic has been ubiquitous and pervasive, there is still little known about how specific groups may be impacted differently. Epidemiological survey research suggests that college students are at heightened risk for negative mental health effects of the pandemic, but most research on collegiate mental health during the pandemic has been focused on the general student body and limited in scope. The current research presents two studies examining impacts of COVID-19 in a large clinical sample of treatment-seeking college students, using data collected through the Center for Collegiate Mental Health. First, examining baseline psychological symptom distress in a pre-COVID and a COVID cohort, few differences were seen between the two groups. Second, for students seeking counseling after COVID-19 onset, reported negative impacts of the pandemic across life areas are presented, along with their associations with psychological symptoms, demographics, and reasons for seeking treatment. Students who reported a negative impact of COVID-19 on their mental health were more likely to present with increased baseline symptom distress. Furthermore, students with minoritized identities were often disproportionately negatively impacted across life areas assessed. Recommendations for clinicians, counseling centers, and institutions are highlighted based on this examination.

From late 2019 or early 2020, communities around the globe have been impacted by the arrival, expansion, and evolution of the coronavirus disease (COVID-19) pandemic. This pandemic has had wide-reaching impacts on public health, economics, travel, social structures, and beyond; mental health is no exception. Over the evolving course of COVID-19, scientists and medical professionals have published myriad calls for research on the short- and long-term mental health effects of COVID-19, both generally (Holmes et al., 2020) and within specific populations like college and university students (Liu et al., 2020; Zhai & Du, 2020). Researchers have responded worldwide, working to answer these questions initially with cross-sectional survey studies or by leveraging ongoing longitudinal projects to address mental health concerns across the continuing timeline of the global crisis.

Several themes from these studies are summarized below; overall, the existing narrative suggests that COVID-19 has negatively impacted mental health both in general population and college student samples. While this may be unsurprising, several as-yet unexplored questions have emerged that the current research attempts to address. Most early studies have understandably been narrow in scope, focusing on (1) a relatively small or homogeneous geographic region, (2) a small subset of psychological symptoms (most often anxiety, depression, and stress, although a variety of problems have been identified, Hossain et al., 2020), and (3) cross-sectional epidemiological data. Existing longitudinal studies tend to focus on a specific subpopulation (e.g., first-year college undergraduates) and examine changes on a relatively short time scale (e.g., beginning to end of the spring 2020 semester). Further, the bulk of published research so far has not examined the mental health impacts of the pandemic on clinical samples of college students seeking psychological services, whose unique mental health needs were already documented prior to the pandemic (Center for Collegiate Mental Health (CCMH), 2016; Xiao et al., 2017). The aims of the current research are twofold: 1) to present a longitudinal comparison of average psychological symptom reports from college student cohorts who sought counseling before and during the COVID-19 pandemic, and 2) to complement and expand the current literature by exploring self-reported negative impacts of COVID-19 in a nationally representative clinical collegiate population.

## Epidemiological mental health impact of COVID-19

The ubiquity of the pandemic has lent itself to epidemiological survey research, allowing researchers to broadly assess communities around the globe. Such research informs an understanding of how mental health may generally change during times of crisis and who may be at increased risk for psychological symptoms. An early systematic review summarizing available research on general population mental health impacts of COVID-19 identified nineteen papers published as late as May 2020 from eight different countries, comprising data from 263 to 52,730 individuals. This review found that the most evident trend in the data was a diversity of symptom experiences: 6.3–50.9% of samples surveyed endorsed high anxiety, 14.6–48.3% indicated depression, 7.0–53.8% had post-traumatic stress symptoms (PTSS), and 8.1–81.9% reported high levels of stress (Xiong et al., 2020). Heterogeneity in findings across studies was also highlighted by a review looking at the pandemic’s mental health impacts on not only the general population, but also specific higher-risk populations like healthcare workers or patients who have contracted COVID-19 (Vindegaard & Benros, 2020).

Part of this diversity in findings both within and across samples could be due to differential impacts of the COVID-19 crisis on subgroups of the population. Subgroups identified most consistently across studies as being at increased risk for psychological symptoms during the pandemic have been women, people with self-reported pre-pandemic mental health conditions, and young people (typically defined as under the age of 25; O’Connor et al., 2020; Pierce et al., 2020; Rossi et al., 2020; Vindegaard & Benros, 2020; Xiong et al., 2020). Additionally, cross-sectional research on individuals in the general population who self-reported a pre-pandemic mental condition indicates that they perceived a compounding negative impact of COVID-19 on their existing psychological symptoms (Gobbi et al., 2020), perhaps especially so for those with a self-reported anxiety or obsessive-compulsive disorder (Miller et al., 2021). In addition to the general subgroup of young people, some studies indicate that student status in particular confers risk for higher psychological distress in response to COVID-19 (Xiong et al., 2020), which has been further explored across fields of research from psychology to economics.

## COVID-19 impact on collegiate mental health

Indeed, studies comprising this diverse literature indicate that college students have experienced broad impacts of COVID-19. In addition to the widespread decision for campuses to send college students home for an extended period of remote learning, some students have had to delay their graduation and others have had offers for a job or internship revoked, contributing to significant academic and career disruption (Aucejo et al., 2020). Furthermore, the impacts of the pandemic and universities’ responses on major issues for students like food and housing insecurity, financial difficulty, and academic concerns are not yet well understood (Lederer et al., 2021). In a qualitative interview study of 195 student participants at a single university, 71% indicated increased stress during the pandemic related to a broad set of concerns, including difficulty concentrating, sleep pattern disruption, decreased social interactions, academic concerns, and fear/worry about the health both of self and loved ones (Son et al., 2020). It is suggested that such widespread impacts of the pandemic for college students contribute to the complex, nuanced picture of collegiate mental health during this time of crisis.

Initial studies seeking to characterize student mental health in the wake of the COVID-19 pandemic typically surveyed college students sampled from general student bodies within single university systems, finding high cross-sectional rates of anxiety (21.3–42.5%), depression (48.0–74.3%), and suicidal thoughts (18.0–63.3%) when compared to expected prevalence (Cao et al., 2020; Kaparounaki et al., 2020; Wang et al., 2020). Researchers who had begun survey studies prior to the onset of COVID-19 were able to assess longitudinal shifts in the mental health particularly of undergraduate first-years from before the pandemic to after its beginning, or from start to end of the spring 2020 semester. In one cohort, students reported increases in externalizing and attention problems over the course of the semester, but not in internalizing problems (Copeland et al., 2021). However, in another comparable cohort, rates of significant internalizing problems increased, from 18.1–25.3% for anxiety and 21.5–31.7% for depression (Fruehwirth et al., 2021). A meta-analysis of 27 studies published between December 2019 and October 2020 with more than 700,000 total college students found that published prevalence rates for internalizing symptoms significantly increased from before March 2020 to after, from 19.0–37.0% for anxiety and from 21.0–54.0% for depression (Li et al., 2021). Thus, research so far suggests that there has been a general increase in mental health problems, particularly anxiety and depression, although there is clearly variability in the point prevalence and longitudinal change of psychological symptoms among the general college student body. This variability could have many causes, including some overarching limitations of the current literature like small and/or homogeneous samples, survey response bias toward those in distress, and different measurement tools and time windows across studies.

Further complicating the state of mental health in college students are findings that different subgroups are impacted differently by the pandemic. Similar to trends in the general population, research has consistently found that women in college are at increased risk for mental health burden due to COVID-19 compared to other gender identity groups (Browning et al., 2021; Kim et al., 2021), and some findings additionally identified sexual and gender minority students as being at greater risk (Fruehwirth et al., 2021). Investigations of negative impacts of the pandemic by racial or ethnic subgroup show mixed results. For college student samples in the United States, various studies indicate that Asian/Asian American students had increased distress (Browning et al., 2021); that White students were more impacted by the pandemic than Black/African American students (Charles et al., 2021); and, even more granular, that White students had greater increases in anxiety but lesser increases in depression when compared to Black/African American students (Fruehwirth et al., 2021). A longitudinal study of Argentinian college students during an extended quarantine phase of the COVID-19 response indicated that students with self-reported prior mental health diagnoses had higher psychological distress early in quarantine that remained constant over follow-up, while students without a mental disorder history saw significant increases in their distress over the quarantine. In both groups, prior self-reported suicidality predicted worse mental health symptoms during quarantine (López Steinmetz et al., 2021). While the trends are complex, investigating the impact of COVID-19 by subgroups contributes to a more nuanced understanding of collegiate mental health and could impact treatment implications for students seeking counseling.

## Current study

Prior to the onset of COVID-19, demand for college counseling services was outpacing increases in enrollment fivefold, which was disproportionately represented by students with threat-to-self characteristics (histories of self-harm and serious suicidal ideation), who utilized 27% more services than students without these tendencies (CCMH, 2016). Moreover, students receiving clinical services were reporting increasing levels of generalized anxiety, depression, social anxiety, family distress and academic distress symptoms when they entered treatment (Xiao et al., 2017). Investigating the health effects of the global COVID-19 pandemic within the clinical population of college students is a clear need given the concerning mental health trends that persisted prior to the onset of COVID-19. During the pandemic, college students encountered unique challenges beyond the developmental and adjustment difficulties that students typically experience, and it is yet unknown how these factors exacerbated the mental health trends that were prevalent before the beginning of the pandemic. Federal and university-level administrative decisions about pandemic responses have had substantial implications for students’ food/housing insecurity, financial difficulty, and academic or career trajectories, which is critical to explore (Lederer et al., 2021). Additionally, requests for research related to epidemiological impacts of COVID-19 stratified by race/ethnicity, gender, age, and prior mental health history (Vigo et al., 2020) remain relevant to increase our understanding of how the pandemic has variably impacted different groups.

Toward these open questions, two studies will be presented here, with the overall goal to comprehensively examine multiple perspectives on COVID-19”s impact on collegiate mental health, using data from a large, nationally representative United States clinical sample of college students seeking psychological services. We hope to clarify some of the heterogeneity in findings that exists so far in the literature by collecting self-reports of a wider variety of psychological symptoms and incorporating analyses of specific subgroups. Study 1 represents a longitudinal comparison of presenting concerns between students who sought treatment either before or during the pandemic and seeks to address the following research question: Did students” presenting concerns, either determined by self-reported psychological symptoms or clinician assessment, differ between “pre-COVID” and “COVID” cohorts? Study 2 summarizes how students who sought treatment after the onset of COVID-19 were impacted by the pandemic, guided by the following research questions:

1. What negative impacts of COVID-19 did students experience?
2. Did students who reported negative mental health impacts of COVID-19 or who sought services due to COVID-19 differ from students who did not?
3. How did students’ self-reported negative impacts of COVID-19 differ by demographic characteristics?

## General methods

### Procedure

The Center for Collegiate Mental Health (CCMH) is well-positioned to explore these research questions. A practice-research network (PRN) based in the United States, CCMH comprises more than 750 college and university counseling center (UCC) members (Castonguay et al., 2011), all of which administer standardized assessment measures in routine practice to clients seeking counseling. UCC members of CCMH who choose to establish and maintain approval from their local Institutional Review Board (IRB) then contribute data from these measures to a centralized repository that is housed and maintained by CCMH staff at the Pennsylvania State University (PSU). Data is only contributed from those clients who have provided informed consent for their treatment data to be used for research purposes. IRB approval for research procedures across the PRN is maintained at PSU. The procedure for the current study was limited to analyses of non-identifiable data collected under the CCMH IRB and did not require additional approval.

### Measures

#### Counseling Center Assessment of Psychological Symptoms (CCAPS)

The CCAPS was developed to serve as a self-report repeated assessment measure with either 62 (Locke et al., 2011) or 34 (Locke et al., 2012) items designed and normed around common concerns for college students; this study uses the CCAPS-62. In addition to an overall metric of psychological distress, the Distress Index (DI), the CCAPS-62 yields eight subscale scores: Depression, Generalized Anxiety, Social Anxiety, Academic Distress, Eating Concerns, Hostility, Family Distress, and Substance Use. In the validation study for the CCAPS-62 (Locke et al., 2011), each of the subscales demonstrated acceptable to very good internal consistency (Cronbach’s alpha from .78 to .91) and acceptable test-retest reliability over periods of one week (*r*_min_ = .78, *r*_max_ = .93) to two weeks (*r*_min_ = .76, *r*_max_ = .92). The current study examined differences in clients’ presenting CCAPS-62 subscale scores based on self-reported impact of COVID-19 and comparisons between the pre-COVID and COVID cohorts.

#### Standardized Dataset (SDS)

The SDS is used to collect demographic, cultural, and mental health history information from UCC clients at the beginning of a treatment course (CCMH, 2022). SDS data in this study were used to gather descriptive information about the samples and, in Study 2, to assess any differences in perceived impact of COVID-19 based on demographic groups.

#### COVID-19 Impact Questions

In July 2020, several questions were added to the SDS to address the COVID-19 pandemic’s impact on students presenting for counseling. Specifically, clients were asked (1) whether their reasons for seeking services were related to the COVID-19 pandemic (yes or no), and (2) which areas of their life had been negatively impacted by COVID-19, including mental health, academics, and relationships (check all that apply; all twelve response options are listed in Table 3). Study 2 examines these impacts and associated client characteristics.

**Table 1.** Characteristics of study 1 cohorts

Variable	Pre-COVID	COVID	
*N*	81,539	51,518	
Age (mean (sd))	21.8 (4.0)	22.2 (4.4)	
Academic class status (n (%))			***
Freshman/First-year	15076 (21.1)	7303 (16.2)	
Sophomore	14851 (20.8)	8532 (18.9)	
Junior	15549 (21.7)	9851 (21.8)	
Senior	14887 (20.8)	9996 (22.1)	
Graduate/Professional degree student	10342 (14.5)	9051 (20.0)	
Non-student	99 (0.1)	70 (0.2)	
High-school student taking college classes	6 (0.0)	2 (0.0)	
Non-degree student	147 (0.2)	60 (0.1)	
Faculty or staff	38 (0.1)	31 (0.1)	
Other (please specify)	528 (0.7)	316 (0.7)	
Race/Ethnicity (n (%))			*
African American/Black	6712 (9.5)	3961 (9.1)	
American Indian or Alaskan Native	375 (0.5)	223 (0.5)	
Asian American/Asian	6141 (8.7)	3693 (8.5)	
Hispanic/Latino/a	6268 (8.9)	4000 (9.2)	
Native Hawaiian or Pacific Islander	148 (0.2)	79 (0.2)	
Multi-racial	3586 (5.1)	2149 (4.9)	
White	46396 (65.6)	28816 (66.1)	
Self-identify	1062 (1.5)	702 (1.6)	
Gender identity (n (%))			***
Woman	45263 (65.4)	29735 (68.2)	
Transgender woman^	0 (0.0)	147 (0.3)	
Man	22143 (32.0)	12444 (28.5)	
Transgender man^	0 (0.0)	204 (0.5)	
Non-binary^	0 (0.0)	748 (1.7)	
Transgender	626 (0.9)	21 (0.0)	
Self-identify	1184 (1.7)	305 (0.7)	
Sexual Orientation (n (%))			***
Asexual^	0 (0.0)	823 (2.0)	
Bisexual	9022 (13.6)	5556 (13.6)	
Gay	1889 (2.8)	1195 (2.9)	
Heterosexual/Straight	49399 (74.3)	28893 (70.5)	
Lesbian	1403 (2.1)	851 (2.1)	
Pansexual^	0 (0.0)	932 (2.3)	
Queer^	0 (0.0)	795 (1.9)	
Questioning	2463 (3.7)	1463 (3.6)	
Self-identify	2271 (3.4)	456 (1.1)	
International student status = Yes (n (%))	3950 (5.8)	2281 (5.2)	***

^These identity options were added to the SDS gender identity and sexual orientation items between data collection for the pre-COVID and COVID cohorts.

**p < .01, ***p < .001.

**Table 2.** Post-hoc ANOVA tests comparing CCAPS-62 Subscales by Cohort

Subscale	Mean(pre-COVID cohort)	Mean (COVID cohort)	*F*(1, 107930)		Cohen’s *d*
Depression	1.82	1.83	2.09		0.01
Eating Concerns	1.06	1.11	88.48	***	0.06
Substance Use	0.62	0.58	66.84	***	−0.05
Generalized Anxiety	1.89	1.90	1.76		0.01
Frustration/Anger	0.96	0.96	0.29		0.00
Social Anxiety	2.08	2.06	8.26	**	−0.02
Family Distress	1.38	1.44	112.02	***	0.07
Academic Distress	1.89	2.04	574.60	***	0.15
Distress Index	1.83	1.86	32.65	***	0.04

** *p* < .005, *** *p* < .001

**Table 3.** Self-reported negative impacts of COVID-19

Area of Impact	Frequency (*n*)	Percent
Mental health	71166	72.3%
Motivation or focus	67901	69.0%
Loneliness or isolation	65601	66.7%
Academics	65305	66.4%
Missed experiences or opportunities	59875	60.9%
Relationships (Significant other, friends, family)	43383	44.1%
Career/Employment	41454	42.1%
Financial	33851	34.4%
Health concerns (others)	29324	29.8%
Health concerns (self)	26301	26.7%
Grief/loss of someone	11421	11.6%
Food or housing insecurity	8346	8.5%
Discrimination/Harassment	2804	2.9%
Other (please specify)	1299	1.3%

#### Clinician Index of Client Concerns (CLICC)

The CLICC is a checklist of 53 concerns that clinicians can complete following a client’s initial appointment (CCMH, 2021). Clinicians are asked to report a) all concerns applicable for a new client and b) of those selected, the client’s primary or top presenting concern. Study 1 investigated whether there were substantial differences in the most frequently identified primary/top presenting concerns between the pre-COVID and COVID cohorts.

## Study 1: Methods

### Sample

Data for Study 1 were collected in Fall 2019 (7/1/2019–12/31/2019; “pre-COVID” cohort) and in Fall 2020 (7/1/2020–12/31/2020; “COVID” cohort). Clients were included who presented for an initial counseling session during either of the two cohort time periods at a participating CCMH center that contributed data during both Fall semesters. Additionally, clients were required to have data for the CCAPS-62 and/or CLICC. After applying these criteria, CCAPS data were available for 102,773 clients from 107 centers (Fall 2019 *N* = 65,653; Fall 2020 *N* = 42,279). CLICC data were available for 70,367 clients from 87 centers (Fall 2019 *N* = 46,077; Fall 2020 *N* = 27,741). Table 1 summarizes client demographic information for the combined sample of clients with CCAPS and/or CLICC data across the two cohorts. For both the pre-COVID and COVID cohorts, clients were predominantly White (65.6–66.1%) and women (65.4–68.2%), with a mean age of 21.8–22.2 years.

### Data analysis

To compare students’ self-reported presenting concerns between the pre-COVID and COVID cohorts, a MANOVA was conducted to examine differences across the nine CCAPS-62 subscales at each client’s baseline (first) administration. Additionally, differences in clinician-reported presenting concerns between the two cohorts were assessed with a Chi-squared test of students’ primary presenting concerns on the CLICC.

## Study 1: Results

The research question for Study 1 asked whether two cohorts of treatment-seeking students initiating counseling either before or after the onset of COVID-19 had different presenting concerns. To investigate this question, analyses first assessed mean self-reported psychological symptoms of clients presenting for an initial session during Fall 2019 (pre-COVID) or Fall 2020 (COVID). MANOVA results indicated that there was a significant difference between the two cohorts when the nine baseline subscale scores were considered as a set, *F*(9,107922) = 132.53, *p* < .001. To further parse apart this result through the exploratory lens of the current research, post-hoc ANOVAs were conducted, and the critical *p* value was adjusted to .005 using a Bonferroni corrected for the 8 tests conducted. Table 2 shows the results of these post-hoc ANOVAs, specifying differences between the two cohorts for several individual subscales that reached statistical significance at the level of .005 but with minimal Cohen’s *d* effect sizes. While the score differences were small, students in the COVID cohort endorsed higher (more severe) scores at baseline on Eating Concerns, Family Distress, Academic Distress, and the overall Distress Index (DI). The COVID cohort also endorsed lower (less severe) scores at baseline than the pre-COVID cohort on Substance Use and Social Anxiety. For some of the subscales (Depression, Generalized Anxiety, and Frustration/Anger), there were no differences between the cohorts. While many subscales had statistically significant differences, none of effect sizes for the subscales reached the threshold for a small effect.

**Figure 1.**
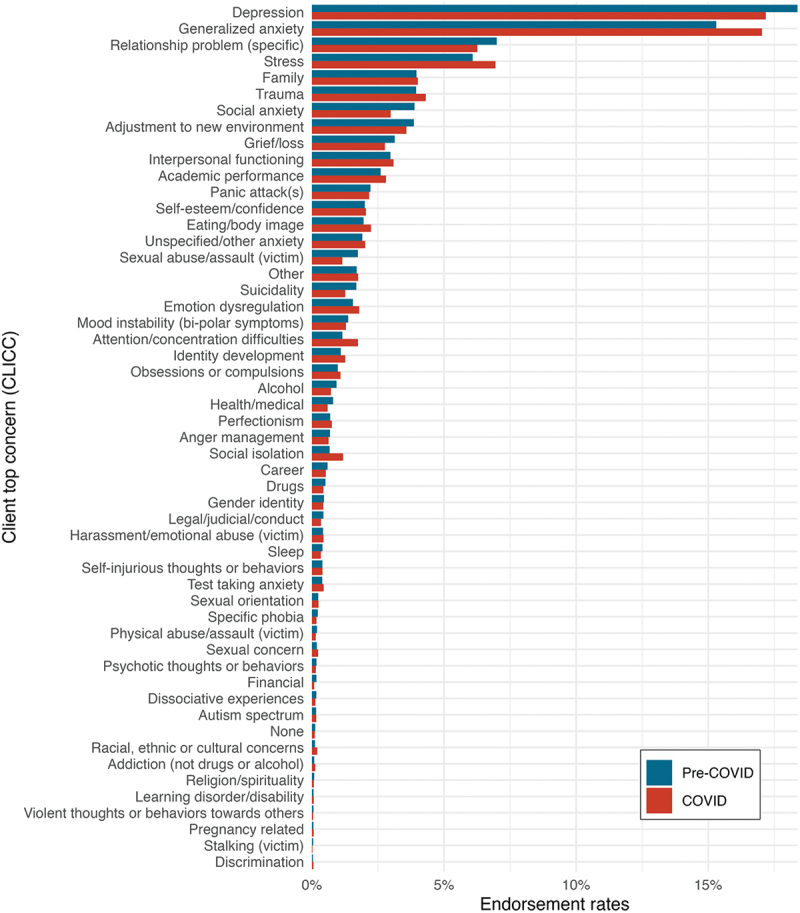
Prevalence of clinician-identified presenting concerns in Fall 2019 and Fall 2020.

**Figure 2.**
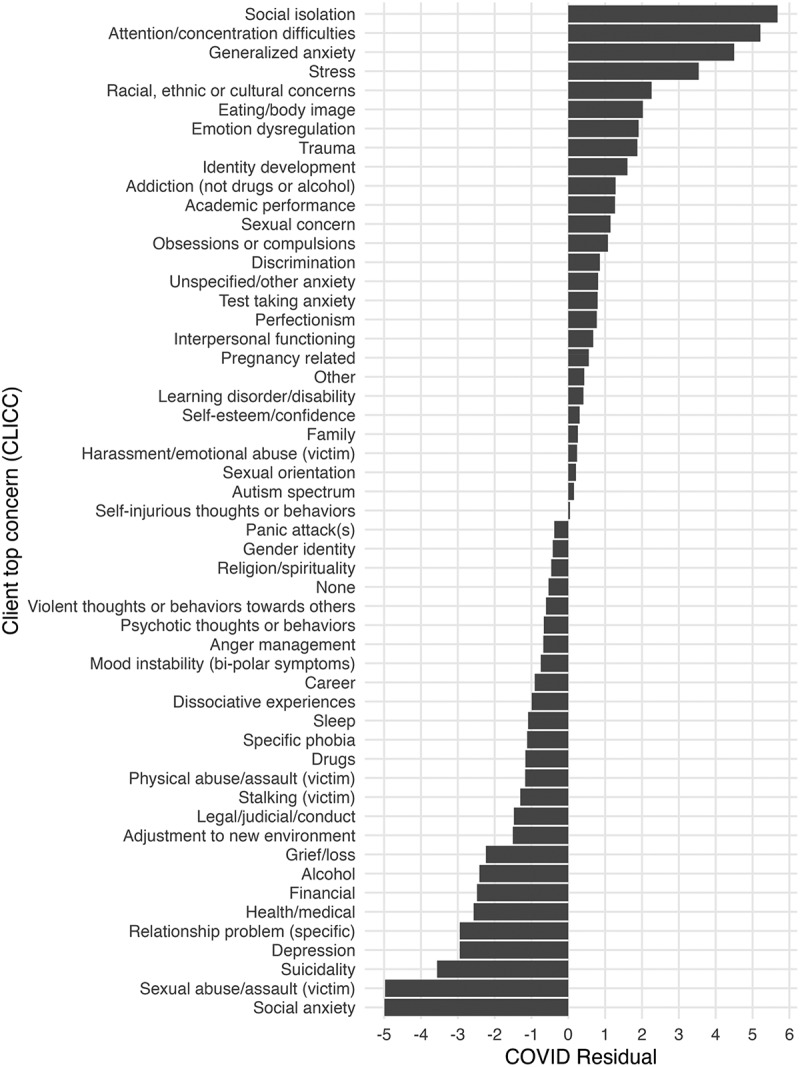
Chi-Squared residuals for clients’ top concerns in COVID cohort.

Additionally, we compared clinicians’ assessment of clients’ primary presenting concerns using the CLICC for the pre-COVID and COVID cohorts. Figure 1 illustrates the relative frequency of clinicians’ identified presenting concerns across the two cohorts. Overall, there were significant differences in the relative frequencies of clients’ primary concerns between the cohorts (*X*^*2*^ = 384.45, *df* = 52, *p* < .001). Figure 2 shows the Chi-squared residuals for the COVID cohort to explore some impactful differences in individual primary presenting concerns between the pre-COVID and COVID cohorts. Social isolation, Attention/concentration difficulties, Generalized anxiety, and Stress were overrepresented as primary concerns per clinician report in the COVID cohort, while Social anxiety, Sexual abuse/assault (victim) and Suicidality were underrepresented as primary concerns compared to the expected values.

## Study 2: Methods

### Sample

Study 2 evaluated the self-reported impact of COVID-19 on a sample of students who initiated treatment after the onset of COVID-19, between 7 January 2020 and 6/30/2021 (the 2020–2021 academic year). Clients were included who presented for an initial counseling session during this period and who completed the COVID Impact Questions. For Study 2, there were 98,371 students seeking treatment at 164 counseling centers. The geographic region of centers contributing COVID data closely matched the geographic breakdown in the broader CCMH membership, with 22.6% of centers in the Midwest, 34.8% of centers in the Northeast, 32.3% of centers in the South, and 10.4% of centers in the West (CCMH, 2023). Demographic information for clients can be found in Appendix Tables A1-A5. Clients were predominantly White (64.1%) and women (67.0%), with relatively even representation across academic class status (first year students were the smallest group, 17.1%, and third year students the largest, 22.6%).

### Data analysis

Descriptive statistics are provided to address the first research question, exploring the frequencies and types of negative impacts of COVID-19 that treatment-seeking college students experienced. To address the second research question of Study 2, clients were classified according to (a) whether they reported seeking counseling because of COVID-19 (*Yes* or *No COVID*), and (b) whether they identified “mental health” as a life area negatively impacted by COVID-19 (*Yes* or *No MH*). To then examine differences in symptom presentation across these groups, a MANOVA was conducted to predict the nine CCAPS-62 subscales as a set by the client groups as classified above, including their interaction, with post-hoc ANOVAs conducted to examine differences by subscale. A Bonferroni correction was applied to adjust for the 8 tests conducted, bringing the critical *p* value to .005. Additionally, partial eta squared (η_p_^2^) effect sizes are reported to convey the proportion of total variation explained by each predictor variable and their interaction after controlling for the other variables. Finally, descriptive statistics are provided to explore differences in COVID-19 negative impacts by various demographic groups.

## Study 2: Results

### Self-reported negative impacts of COVID-19

The first research question of Study 2 explored the negative impacts of COVID-19 reported by treatment-seeking students. First, students seeking services were asked whether their reasons for doing so were related to the COVID-19 pandemic. Of those who responded to this question (*n* = 93,877), 32.0% indicated that their reasons for seeking counseling or psychological services were related to the pandemic. All respondents to the COVID Impact Questions (*n* = 98,371), regardless of whether their reasons for seeking services were related to COVID-19, were also asked to report which areas of their lives had been negatively impacted by the pandemic. Table 3 presents the proportions of clients who indicated an impact in each of twelve areas; overall, 94% of clients reported that at least one area was negatively impacted, while 90% reported negative impacts in multiple life areas. The most frequently endorsed areas of impact were mental health (73.2%), motivation or focus (69.0%), loneliness or isolation (66.7%), academics (66.4%), and missed experiences or opportunities (60.9%).

**Figure 3.**
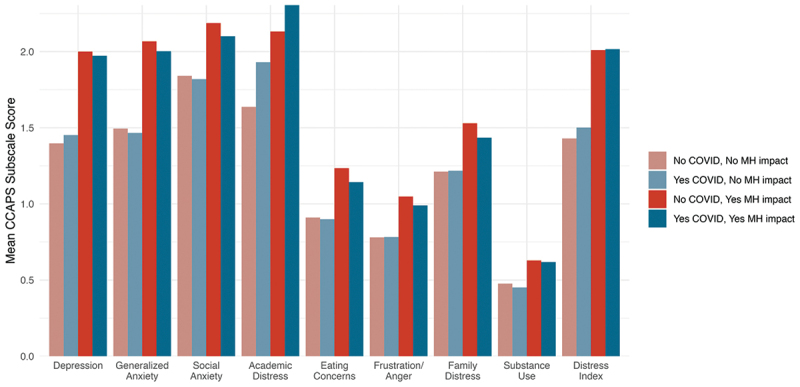
CCAPS subscale scores for clients reporting mental health impact of COVID-19.

*Note*: No COVID-19 corresponds to client indicating that their reasons for seeking services were not in any way related to the COVID-19 pandemic and related events. No MH impact indicates they did not report mental health as a negative impact of COVID-19.

MANOVA results addressing the second research question of differences between clients based on their reported impact of COVID-19 indicated significant differences in baseline CCAPS-62 subscale scores as a set by (a) seeking services due to COVID-19 *(Yes* vs. *No COVID*; *F*(9,70802) = 230.38, *p* < .001), (b) having a negative mental health impact of COVID-19 *(Yes* vs. *No MH*; *F*(9, 70802) = 964.89, *p* < .001), and the interaction of these two variables, *F*(9,70802) = 6.91, *p* < .001. Results from post-hoc ANOVA tests are detailed in Table 4. Students who reported seeking treatment due to COVID-19 *(Yes COVID*, 32% of respondents) endorsed higher baseline subscale scores on Depression, Substance Use, Generalized Anxiety, Academic Distress, and the DI. Regardless of their reasons for seeking services, students who reported a negative mental health impact of COVID-19 (*Yes MH*, 73.2% of respondents) also reported higher levels of distress on all nine CCAPS-62 subscales. Finally, the interaction between *Yes COVID* and *Yes MH* was statistically significant for Depression, Eating Concerns, Frustration/Anger, Social Anxiety, Family Distress, Academic Distress, and the DI. For Depression, Frustration/Anger, and Family Distress, there were opposite effects of seeking services due to COVID-19 for clients who did and did not report mental health impacts of COVID-19. Clients who reported no mental health impact (*No MH*) reported higher CCAPS scores associated with seeking services due to COVID-19 (*Yes COVID*), but when clients did report a mental health impact (*Yes MH*), seeking services due to COVID-19 (*Yes COVID*) was associated with lower CCAPS scores in those domains. For Eating Concerns and Social Anxiety, the effect of seeking services due to COVID-19 was larger for clients who also reported negative mental health impacts. Academic Distress showed a unique pattern of scores across the four groups, such that seeking services due to COVID-19 was associated with higher scores in both clients who did and did not report mental health impacts of COVID-19. In other words, clients who reported both seeking services due to COVID-19 and having mental health impacts also endorsed the highest level of Academic Distress. Figure 3 shows these interactions.

**Table 4.** Post-hoc ANOVA tests exploring presenting concerns by yes COVID and Yes MH

Subscale	*Yes COVID* (vs. *No COVID*)			*Yes MH* (vs. *No MH*)			Interaction		
	*F*	η_p_^2^	*p*	*F*	η_p_^2^	*p*	*F*	η_p_^2^	*p*
Depression	291.23	<.01	***	6610.21	.09	***	19.40	<.01	***
Eating Concerns	2.06	<.01		1593.12	.02	***	16.56	<.01	***
Substance Use	12.25	<.01	***	559.58	<.01	***	0.79	<.01	
Generalized Anxiety	85.16	<.01	***	5557.56	.07	***	3.47	<.01	
Frustration/Anger	1.66	<.01		1404.45	.02	***	11.92	<.01	***
Social Anxiety	0.09	<.01		1814.04	.02	***	10.32	<.01	**
Family Distress	2.35	<.01		1195.66	.02	***	20.57	<.01	***
Academic Distress	1496.23	.02	***	3318.57	.04	***	32.88	<.01	***
Distress Index	510.20	<.01	***	7915.17	.10	***	23.74	<.01	***

All values displayed are *F*(1, 70810). *Yes COVID* represents the group of students whose reasons for seeking treatment were related to COVID-19, while the *No COVID* group was not seeking treatment for COVID-19-related reasons. *Yes MH* represents the group of students whose mental health was negatively impacted by COVID-19, while the *No MH* group did not report a negative mental health impact of COVID-19.

** *p* < .005, *** *p* < .001.

### Demographic subgroup analyses

Finally, the third research question of Study 2 explored whether there were differences in endorsement rates of the twelve self-reported negative impact areas of COVID-19 listed in Table 3 by subgroups within several demographic grouping characteristics: academic class status, race/ethnicity, gender identity, sexual orientation, and international student status. Demographic data were collected through the SDS, which has a standardized set of response options. The endorsement rates for each interaction of the twelve negative impacts and the demographic characteristic subgroups/identities are detailed in Appendix Tables A1-A5; some of the main findings are highlighted here.

#### Academic class status

Comparing reported negative impacts of COVID-19 by students’ academic class (Table A1), results indicated that Freshman/First-year undergraduate students reported the highest rates of COVID-19 negative impact on mental health (75.6%), loneliness or isolation (71.3%), and missed experiences or opportunities (69.8%). Senior undergraduates, on the other hand, endorsed the highest rate of negative impact in the areas of career/employment (51.7%) and finances (41.2%).

#### Race and ethnicity

While the results comparing rates of endorsed negative impacts by race/ethnicity subgroups are complex (Table A2), several findings stand out. Students who identified as American Indian or Alaskan Native reported the highest rates of COVID-19 negative impact in the areas of finances (52.5%), personal health concerns (34.7%), grief/loss of someone (23.9%), and food/housing insecurity (19.3%). Compared to students with any other racial/ethnic identity, those who identified as African American/Black reported the lowest rates of negative impact in many areas, including mental health (62.8%), motivation (65.3%), loneliness (59.5%), academics (63.2%), missed experiences (50.4%), relationships (40.3%) and health concerns for others (23.5%). Students identifying as Asian American (10.1%) or Native Hawaiian or Pacific Islander (8.6%) reported the highest rates of discrimination/harassment. Students who identified as Native Hawaiian or Pacific Islander indicated the greatest rate of negative impact on academics (73.7%) and relationships (52.2%). Finally, students identifying as White reported the highest rates of missed experiences or opportunities (64.3%) but the lowest rates of health concerns for self (25.5%), grief/loss of someone (10.2%), food or housing insecurity (7.1%), and discrimination/harassment (1.0%).

#### Gender identity

As illustrated in Table A3, results comparing negative impacts by students’ reported gender identity indicate that those who identify as transgender men, non-binary, or who self-identified reported higher rates of COVID-19 negative impact in almost every domain when compared to the overall rates and all other gender identity groups, with non-binary students endorsing the highest rates of impact in most areas.

#### Sexual orientation

Results of the comparison by sexual orientation identity (Table A4) indicated that students identifying as pansexual, queer and questioning endorsed the highest rates of negative impacts from COVID-19 in many areas, while heterosexual/straight and asexual students reported the lowest rates of negative impacts.

#### International student status

Interestingly, when comparing international students to domestic students, international students reported lower rates of COVID-19 negative impact in nearly all areas (Table A5). A notable exception is in discrimination/harassment, where international students (6.2%) reported more than twice the rate of domestic students (2.6%).

## General discussion

Comparing longitudinally across cohorts of students presenting for counseling, Study 1 found small differences in presenting concerns, both by client and clinician report, for students seeking treatment in the fall 2020 semester (COVID cohort) and those from the fall 2019 semester (pre-COVID cohort). On average, COVID cohort clients self-reported statistically higher distress in the areas of Eating Concerns, Family Distress, and Academic Distress, although the effect sizes for these differences were minimal to small. Additionally, from pre-COVID to COVID, the largest increases in prevalence of clinician-identified presenting concerns were seen in problem areas of Stress, Social isolation, Generalized anxiety, Academic Performance, Attention/concentration difficulties, Eating/body image, Sleep, Trauma, and Family. While the effect sizes from analyses of client-reported presenting concerns limit the conclusions that may be drawn, in the exploratory lens of this research it is worth noting the overlap in heightened presenting concerns from both client and clinician perspective in the COVID cohort (Academics, Eating/body image concerns, and Family). A noticeable difference between client and clinician reports, however, was the increase in clinician-identified Anxiety/Generalized anxiety without an apparent increase in students’ self-reported scores on the Generalized Anxiety subscale of the CCAPS. In seeking to understand this difference, we examined item-level differences on the CCAPS between the pre-COVID and COVID cohorts and found that several of the specific symptoms contributing to the Generalized Anxiety subscale had increased (being easily frightened, racing thoughts, general fear, and tension), but that these increases were offset by decreases in other symptoms, such as fear of having panic attacks in public (a situation many were unlikely to encounter due to widespread shelter-in-place and social distancing mandates).

While these increases, particularly in stress, social isolation, and anxiety, are consistent with prior reports of collegiate mental health impacts of COVID-19 (Li et al., 2021; Miller et al., 2021; Wang et al., 2020), a marked difference is that our results indicated consistency in presenting concerns from pre-COVID to COVID cohorts for many problem areas, including Depression, Suicidal Ideation, and General Distress. Indeed, even psychological subscales showing a statistically significant difference between cohorts had minimal effect sizes (Eating Concerns and Family Distress). One likely reason for this contrast with existing literature is that while prior research has examined mental health in broad samples of college students generally, the current work is focused on clinical samples of college students seeking treatment for their mental health concerns. Using depression as an example, approximately 50% of clients in both the pre-COVID and COVID cohorts had depression identified by their clinician as a presenting concern. While the prevalence within these clinical samples did not change, there may be a kind of “ceiling effect” occurring because the prevalence of clients with a depression presenting concern was already high before the onset of COVID-19. From the current analyses, the strongest (though still small) effect seen was that both clients and clinicians indicated higher rates of academic concerns for the COVID cohort.

Study 2 analyses focused only on students seeking treatment in the wake of COVID-19, on the other hand, found that the vast majority of students presenting for counseling in the 2020–2021 academic year self-reported negative impacts of the pandemic on diverse areas of their lives (94%), not just in academics. For many (72%), these negative impacts included their mental health, and 33% reported seeking treatment for COVID-19-related reasons. Students who reported a negative impact of COVID-19 on their mental health also tended to self-report higher severity on average across areas of distress than students who indicated that their mental health was not affected by the pandemic. This seems to suggest that in this population, the phrase “mental health” is being used to capture a wide array of psychological problems and heightened distress for these students. Additionally, self-reported Academic Distress seems to be uniquely elevated for students seeking services because of COVID-19, those who reported that their mental health had been negatively impacted, and the interaction of these two items. This suggests that increased Academic Distress is being influenced by both environmental adjustments to the pandemic (e.g., remote learning environments, living at home) and students’ perceptions and experiences of their own mental health. In conjunction with the findings from Study 1, it seems that academic distress and performance is a uniquely important contributor to students seeking mental health counseling during the COVID-19 pandemic, which may reflect the breadth of services available in counseling centers along with the breadth of distress students may experience as a result of their academics.

While academics stand out across both studies as consistently elevated for students seeking treatment during the pandemic, other sets of results from Study 1 and Study 2 might seem contradictory. It is important to remember that the comparison being drawn in each study is different. In Study 1, results indicated that students presenting for counseling during the pandemic reported similar symptom severity on the CCAPS-62 when compared to students who had presented counseling prior to COVID-19. However, in Study 2, these students seeking treatment during COVID-19 reported negative impacts of the pandemic on their mental health and many other domains, comparing introspectively to their own experience before the pandemic. In contextualizing this difference further, results from the CCMH 2021 Annual Report (CCMH, 2022) suggest that beyond presenting clinical symptoms, several mental health trends shifted during the wake of the COVID-19 pandemic. Most notably, rates of prior treatment (counseling, medication, hospitalization) decreased after years of consistent increases. Additionally, rates of reported threat-to-self indicators decreased during 2020–2021 after consistently rising over the prior eight years. CCMH suggested that these shifts might be explained by a change in the type of student who tended to seek treatment at UCCs during the pandemic compared to pre-COVID (CCMH, 2022). For example, trends they highlight include seeing a greater percentage of students who hadn’t sought counseling before, students who wouldn’t have been able to utilize in-person services but who were able to be seen due to the rise in virtual counseling, and fewer students with prior mental health treatment histories who presumably had access to these resources when they lived off-campus during remote learning periods. Thus, there may be factors beyond those assessed in the current research representing differences between students seeking treatment before or during the COVID-19 pandemic.

Finally, analyses examining the third research question of Study 2, which sought to investigate different patterns across COVID-19 impacts by demographic groups, were broad and complex. However, if there is one clear result across these analyses, it is that mental health was consistently the most reported negative impact of COVID-19 across 36 of 38 demographic identities, although only 33% of students in this sample sought mental health services because of COVID-19. The only exceptions to this trend were among African American students and students who self-identified their race/ethnicity, who identified “motivation or focus” as the most common concern, followed by mental health. The consistency of this finding across demographic groups indicates that universities and counseling centers should be prepared to support the mental health needs of nearly all students as the impacts of COVID-19 continue.

Across the specific demographic characteristics examined, it is important to note that both large and small differences were discovered between the various identity groups, but the lowest rates of negative impact were often associated with majority identity statuses. There is no question that COVID-19 is profoundly impacting all students. With that being said, the data reviewed here highlight that the negative impacts of COVID-19 are often reported disproportionally by students with minoritized identities. This disproportionate impact is also likely to be heightened for students with multiple intersecting minoritized identities. For example, 62.8% of students who identified as African American/Black reported that their mental health was negatively impacted due to COVID-19, but this rate significantly increased to 72.9% if those students also identified as bisexual. While further research will be needed to fully comprehend the nuanced relationship between COVID-19 and various intersecting identities, colleges and universities are encouraged to consider how the findings presented here can be used to inform programming and support services at their campus. Additionally, results from the Academic Status variable analysis indicate developmental differences in that first-year students reported coping with more significant impacts of missed experiences than their peers, while fourth-year students reported the highest level of impact in career/employment, as well as elevated impacts in the areas of finances and food/housing. While these analyses were complex, it should be clear that the COVID-19 pandemic has impacted everyone, but the extent of its negative impacts on this clinical population tended to vary by individual client characteristics.

### Implications

Even after years of living with the changes wrought by the COVID-19 pandemic, we are still only beginning to understand its impact and the ways that help-seeking college students have been affected. One trend evident in the current sample that may have far-reaching implications is that those students who self-report that their mental health has worsened were also found to self-report higher severity of distress across a wide array of psychological problems. When students broadly perceive negative mental health impacts from COVID-19, this should alert clinicians that these individuals are likely to concurrently be struggling with a variety of difficulties and symptoms. Another clear finding is that treatment-seeking students are reporting numerous negative impacts of COVID-19 in addition to mental health, including academic concerns, grief/loss, missed opportunities, and basic needs (financial, food or housing insecurity). Furthermore, people belonging to diverse identity groups have been disproportionately impacted by the pandemic. While the focus of the current research was on the COVID-19 pandemic, these findings must also be put in context of the larger sociopolitical landscape of the United States, including a concurrent crisis of racial injustice that also had a disproportionate impact on marginalized communities (Moffitt et al., 2022; Sanchez et al., 2022). Clinicians should be prepared to incorporate these findings in their practice when they are assessing student clients. This involves expanding their clinical assessments to cover the comprehensive impacts discovered in this study, including psychological symptoms, developmental and psychoeducational concerns, psychosocial stressors, and identity factors.

Additionally, clinicians, counseling center administrators, and university and college leaders alike should be aware of developmental differences found in this study by students’ academic status. Students earlier in their undergraduate program, particularly first years, reported coping with more significant impacts of missed experiences than their peers, which could have institutional consequences for years to come. Indeed, the COVID-19 pandemic has disrupted the typical opportunities available not just to college or university students, but all those who may enter an institute of higher education in the future. Institutional programming designed to provide entering classes and other early-career undergraduate students additional experiences on campus and to connect with their peers may help students to recuperate some of these losses. Furthermore, older undergraduates, particularly fourth years, were more likely to report grappling with concerns beyond their degree program, like employment, finances, and food/housing insecurity. Thus, clinicians and administrators should be prepared to readily match students with resources that may alleviate stressful concerns for students preparing to graduate.

### Limitations and future directions

While this study has considerable strengths in its ability to capture a variety of impacts of the COVID-19 pandemic across a nationally representative sample of treatment-seeking college students, there are also limitations to consider. First, this research was conducted within naturalistic counseling settings. This means that the administration of questionnaires may not be standardized across separate counseling centers and there were likely changes in counseling center operation and utilization as a function of the COVID-19 pandemic that we were unable to systematically control or measure in this research. Second, it is important to recognize that statistically significant findings presented throughout this research should be seen in the context of their small effect sizes. Interpretation of these results should be conducted cautiously as the findings may not represent clinically meaningful differences. Third, given what little was known about the mental health impacts of the pandemic at the time this research was begun, all analyses presented here were exploratory, which limits the conclusions that we were able to draw. That said, there are several questions raised by results of this study that will be important for future research. One clear trend is that the mental health impacts of COVID-19 on college students are diverse and pervasive; while universities have largely returned to “normal” operations, the pandemic is ongoing and its long-term impacts are yet unknown, for students yet to matriculate, those currently in higher education, and those who have graduated. It will also be important to empirically evaluate the efficacy of programs instituted by universities and counseling centers toward addressing impacts of the pandemic on their students.

## Conclusion

Data from a large, nationally representative clinical sample of students seeking treatment at university and college counseling centers (UCCs) illustrate that this population has been negatively impacted by COVID-19 in many ways. Differences in presenting concerns between pre-COVID and COVID cohorts were few, and it is possible that the cohorts may represent different kinds of treatment-seeking students. Interestingly, those students who report that COVID-19 has affected their mental health also report increased distress in nearly all areas, indicating that “mental health” may function as an internal barometer that encompasses a wide variety of concerns. A clear trend in the literature and this research is that the pandemic has disproportionately affected those with minoritized identities, and this study could characterize some of these differences that counseling center clinicians are likely to encounter. In all, this paper represents a first exploratory examination into a clinical student population, with findings that are readily generalizable to UCCs across the United States.

## Data Availability

Raw data were generated by the Center for Collegiate Mental Health (CCMH). Derived data supporting the findings of this study are available from the corresponding author (NP) on reasonable request.

## Geolocation Information

Data were collected in university and college counseling centers across the United States.

## Appendix

**Table A1.** Endorsement rates of negative impacts of COVID-19 by Academic Class Status

COVID Impact	Freshman/First-year	Sophomore	Junior	Senior	Graduate/Professional degree student
*n* per group (% of total *N*)	15438 (17.1%)	17118 (19.0%)	20394 (22.6%)	19227 (21.3%)	17963 (19.9%)
Mental health	75.6%	76.2%	74.1%	71.1%	67.0%
Motivation or focus	70.6%	71.6%	70.4%	68.7%	65.8%
Loneliness or isolation	71.3%	69.2%	66.1%	64.5%	64.1%
Academics	69.4%	72.3%	68.8%	65.4%	59.2%
Missed experiences or opportunities	69.8%	61.0%	60.2%	60.2%	55.5%
Relationships (Significant other, friends, family)	42.3%	44.9%	44.8%	44.4%	44.0%
Career/Employment	31.6%	38.2%	45.4%	51.7%	42.4%
Financial	28.9%	33.9%	38.4%	41.2%	29.9%
Health concerns (others)	28.6%	28.2%	29.2%	30.4%	32.5%
Health concerns (self)	24.4%	26.2%	27.3%	28.1%	27.6%
Grief/loss of someone	11.1%	11.4%	11.7%	11.7%	12.0%
Food or housing insecurity	6.1%	8.3%	10.1%	10.4%	6.9%
Discrimination/Harassment	2.2%	2.5%	2.6%	3.0%	3.4%
Other (please specify)	1.0%	1.1%	1.2%	1.4%	1.8%

**Table A2.** Endorsement Rates of Negative Impacts of COVID-19 by Race and Ethnicity

COVID Impact	African American/ Black	American Indian or Alaskan Native	Asian American/Asian	Hispanic/ Latino/a	Native Hawaiian or Pacific Islander	Multi-racial	White	Self-identify
*n* per group (% of total *N*)	8302 (9.1%)	461 (0.5%)	9007 (9.9%)	8900 (9.7%)	186 (0.2%)	4544 (5.0%)	58579 (64.1%)	1452 (1.6%)
Mental health	62.8%	75.7%	68.1%	71.0%	74.2%	73.7%	74.3%	70.2%
Motivation or focus	65.3%	70.9%	66.4%	70.5%	68.3%	72.7%	69.2%	71.1%
Loneliness or isolation	59.5%	64.2%	63.0%	64.2%	66.1%	68.9%	68.4%	68.5%
Academics	63.2%	69.0%	63.6%	66.0%	73.7%	67.9%	67.1%	67.1%
Missed experiences or opportunities	50.4%	56.0%	53.9%	55.2%	60.2%	61.7%	64.3%	59.9%
Relationships (Significant other, friends, family)	40.3%	42.7%	43.1%	44.3%	52.2%	45.9%	44.1%	47.9%
Career/Employment	40.3%	45.3%	37.8%	42.2%	41.4%	44.6%	43.0%	43.1%
Financial	42.9%	52.5%	26.4%	43.7%	42.5%	39.1%	32.5%	37.3%
Health concerns (others)	23.5%	31.7%	25.9%	30.1%	29.6%	32.7%	30.8%	34.6%
Health concerns (self)	26.2%	34.7%	28.8%	28.5%	31.2%	29.2%	25.5%	33.5%
Grief/loss of someone	17.2%	23.9%	10.5%	13.9%	19.4%	12.7%	10.2%	14.2%
Food or housing insecurity	12.6%	19.3%	7.2%	11.5%	13.4%	11.3%	7.1%	12.7%
Discrimination/Harassment	5.7%	4.1%	10.1%	2.6%	8.6%	5.0%	1.0%	4.6%
Other (please specify)	1.1%	1.5%	1.1%	1.1%	0.5%	1.3%	1.3%	1.4%

**Table A3.** Endorsement rates of negative Impacts of COVID-19 by gender identity

COVID Impact	Woman	Transgender woman	Man	Transgender man	Non-binary	Self-identify
*n* per group (% of total *N*)	60817 (67.0%)	294 (0.3%)	26774 (29.5%)	409 (0.5%)	1820 (2.0%)	597 (0.7%)
Mental health	74.1%	71.8%	67.2%	79.2%	85.4%	79.7%
Motivation or focus	70.5%	63.9%	64.8%	75.6%	78.8%	74.4%
Loneliness or isolation	67.0%	67.0%	64.9%	73.8%	79.5%	76.7%
Academics	66.1%	68.7%	66.6%	72.9%	76.8%	70.0%
Missed experiences or opportunities	62.8%	54.4%	56.0%	67.5%	70.8%	64.8%
Relationships (Significant other, friends, family)	43.4%	40.5%	44.8%	45.2%	49.7%	51.3%
Career/Employment	41.7%	41.5%	42.4%	44.7%	51.8%	44.4%
Financial	35.2%	36.1%	32.4%	43.0%	45.9%	38.5%
Health concerns (others)	31.1%	32.7%	25.7%	38.9%	44.4%	44.6%
Health concerns (self)	26.8%	31.0%	25.3%	32.3%	38.6%	35.5%
Grief/loss of someone	12.1%	9.9%	10.2%	8.1%	14.8%	13.4%
Food or housing insecurity	8.2%	12.2%	8.2%	15.4%	17.0%	14.4%
Discrimination/Harassment	2.8%	5.1%	2.5%	6.8%	6.3%	6.2%
Other (please specify)	1.2%	3.4%	1.4%	1.7%	1.9%	2.5%

**Table A4.** Endorsement rates of negative impacts of COVID-19 by Sexual Orientation

COVID Impact	Asexual	Bisexual	Gay	Heterosexual/ Straight	Lesbian	Pansexual	Queer	Questioning	Self-identify
*n* per group (% of total *N*)	1792 (2.1%)	11411 (13.2%)	2529 (2.9%)	61113 (70.7%)	1786 (2.1%)	2039 (2.4%)	1876 (2.2%)	3158 (3.7%)	792 (0.9%)
Mental health	69.1%	81.7%	76.3%	69.5%	80.5%	82.8%	84.2%	80.6%	74.2%
Motivation or focus	65.9%	75.8%	71.3%	66.8%	73.9%	77.1%	79.0%	78.0%	69.3%
Loneliness or isolation	61.7%	74.1%	70.5%	64.4%	72.5%	77.0%	77.5%	76.0%	71.0%
Academics	68.0%	72.6%	67.6%	64.5%	69.3%	73.9%	72.7%	73.2%	66.4%
Missed experiences or opportunities	55.8%	67.9%	62.2%	59.1%	65.6%	67.5%	69.6%	70.5%	61.9%
Relationships (Significant other, friends, family)	38.4%	46.8%	48.6%	42.8%	46.2%	50.9%	51.9%	51.3%	50.1%
Career/Employment	40.3%	47.9%	44.6%	40.3%	43.5%	52.1%	51.0%	44.6%	43.8%
Financial	33.0%	41.4%	37.5%	32.2%	39.5%	48.9%	41.8%	35.8%	37.2%
Health concerns (others)	28.3%	36.8%	27.9%	27.3%	35.3%	42.8%	44.6%	36.8%	33.8%
Health concerns (self)	27.5%	31.5%	28.1%	24.8%	30.3%	35.0%	36.9%	33.2%	29.4%
Grief/loss of someone	12.8%	13.1%	10.8%	10.9%	10.5%	15.3%	15.1%	11.8%	12.2%
Food or housing insecurity	9.1%	11.9%	10.0%	7.0%	11.0%	17.5%	14.3%	10.3%	10.9%
Discrimination/Harassment	3.4%	3.3%	4.2%	2.4%	3.2%	5.4%	6.6%	3.1%	3.3%
Other (please specify)	1.6%	1.2%	1.1%	1.2%	1.3%	1.7%	1.7%	1.1%	2.9%

**Table A5.** Endorsement rates of negative impacts of COVID-19 by international student status

COVID Impact	No	Yes
*n* per group (% of total *N*)	85543 (94.0%)	5493 (6.0%)
Mental health	73.0%	60.6%
Motivation or focus	69.6%	59.8%
Loneliness or isolation	67.1%	59.4%
Academics	67.0%	59.4%
Missed experiences or opportunities	62.0%	45.5%
Relationships (Significant other, friends, family)	44.1%	41.7%
Career/Employment	42.6%	36.9%
Financial	34.9%	29.1%
Health concerns (others)	30.3%	24.5%
Health concerns (self)	26.7%	27.7%
Grief/loss of someone	11.7%	10.2%
Food or housing insecurity	8.4%	9.6%
Discrimination/Harassment	2.6%	6.2%
Other (please specify)	1.3%	1.7%
