# Correction

**DOI:** 10.1080/28324765.2025.2457911

**Published:** 2025-01-28

**Article title**: Impacts of the COVID-19 pandemic on treatment-seeking college students

**Authors**: Natalie R. Pottschmidt, Rebecca A. Janis, Brett E. Scofield, Alaina L. Cummins, Dever M. Carney, Katherine A. Davis, J. Ryan Kilcullen, Hongjun (Michael) Tan, Louis G. Castonguay and Benjamin D. Locke

**Journal**: Cogent Mental Health

**DOI**: 10.1080/28324765.2023.2211633

This article was originally published with an error in the text. To rectify this, the following additional content has now been included in the procedure section under the General Methods.

Data is only contributed from those clients who have provided informed consent for their treatment data to be used for research purposes.

